# Effects of the vegetative propagation method on juvenility in *Robinia pseudoacacia* L*.*

**DOI:** 10.48130/FR-2022-0017

**Published:** 2022-12-05

**Authors:** Jie Liu, Zijie Zhang, Yapeng Li, Juan Han, Huayu Si, Yueqi Mi, Shaoming Wang, Xiaoning Wei, Hao Yang, Yuhan Sun, Yun Li

**Affiliations:** 1 Beijing Advanced Innovation Center for Tree Breeding by Molecular Design, Engineering Technology Research Center of Black Locust of National Forestry and Grassland Administration, National Engineering Laboratory for Tree Breeding, College of Biological Sciences and Technology, Beijing Forestry University, Beijing, People's Republic of China; 2 State-Owned Quanbaoshan Forestry Station in Luoning County of He’nan Province, Luoyang, People's Republic of China; 3 State-Owned Lvcun Forestry Farm in Luoning County of He’nan Province, Luoyang, People’s Republic of China; 4 Xiaoxian Forestry Development Center of An’hui Province, Suzhou, People's Republic of China

**Keywords:** Rejuvenation, Cyclophysis, Topophysis, *in situ* root sprouting, Root cutting

## Abstract

Vegetative propagation is an important method of reproduction and rejuvenation in forestry. The growth and development of asexually propagated trees are influenced by the age and position of the propagule on the plant, effects referred to as cyclophysis and topophysis, respectively. Due to the long lifespans and large body sizes of woody trees, the selection of propagules is critically important. Here, we used three vegetative propagation methods (shoot cutting, root sprouting, and root cutting) to study the effect of different regeneration methods on juvenility of the resulting black locust plants, with seed-derived seedlings used as a control. Most characteristics of plantlets generated by root-sprouting were similar to those of seed-derived seedlings, including leaf traits and leaf anatomical structure. However, there were significant differences between the plantlets derived from shoot-cuttings and seedlings from seeds. Furthermore, the data showed that some of these age-related small RNAs and genes differed in expression among propagation methods and between plantlets/seedlings and mature trees. These age-related small RNAs, genes, and transcription factors may be used as molecular markers of juvenility and phase transitions in black locust. Our results provide useful information for the optimal propagation of woody trees and for further research into the mechanisms of root regeneration.

## INTRODUCTION

Black locust (*Robinia pseudoacacia* L., Fabaceae) is a fast-growing tree species from the legume family native to south-eastern North America. Since its introduction to China in the middle of the 19^th^ century, it has become an important ecological afforestation pioneer tree species and is widely cultivated^[1,2]^. Its fibrous roots can range from shallow to deep, depending on the soil type, and are wide spreading and highly adventitious. The roots can produce suckers, especially after being cut or damaged, thus creating dense thickets of new growth. This robust root system is beneficial for soil and water conservation and environmental restoration. Black locust is also a source of wood, fodder, honey, bio-oil, and biomass^[3,4]^. Because of its important role in restoration and commodity production, there is increased interest in breeding and reproductive strategies for black locust.

*R. pseudoacacia* is a versatile landscape tree and is increasingly grown as a horticultural species because of its ornamental attractiveness. Currently, black locust is propagated primarily by seed, root sprouting, and shoot cutting^[5−7]^. Reproduction *via* seeds generates large numbers of progeny with the combined characteristics of both parents in a cost-effective manner, whereas vegetative propagation is useful in clonal forestry for rapid multiplication of superior genotypes (e.g., elite trees) and maintenance of specific desired characteristics. Regardless of the use for black locust propagation, its regeneration potential can decline as the age and maturity of the propagules increase, phenomena referred to as cyclophysis and topophysis, respectively. Juvenile plants often reproduce readily, whereas reproduction may become almost impossible in old trees. Likewise, seeds of young trees show higher germination percentages than those of old trees^[7−10]^. Recent research on the mechanisms that govern plant growth and development has made it increasingly clear that rejuvenation can be achieved by artificial methods^[11−13]^. Plants that reach the adult or reproductive stage can regain their juvenile characteristics through rejuvenation^[14]^, and repeated grafting, serial root cutting, and other methods are useful for rejuvenating such plants^[12,15−17]^. Rejuvenation techniques are therefore extremely important for the success of vegetative propagation.

Plants that reach the adult or reproductive stage can regain their juvenile characteristics through rejuvenation. Juvenile plants have many advantages in terms of growth and vigor that are of importance for their use, especially for perennial woody plants^[18]^. Numerous studies have examined developmental phase transitions in plants, and reported changes in many phase-specific traits such as leaf size and shape, photosynthetic capacity, adventitious rooting potential, and reproductive competence^[19−21]^. Old-growth conifers show significant differences in needle anatomical characteristics compared with younger trees, including longer needles, larger resin canals, smaller vascular cylinders, thinner cell walls, and fewer hypodermal cells^[12,22]^.

Plant rejuvenation refers to the reversal of programmed plant development whereby adult plants regain some or all of their juvenile characteristics. The initiation, progress, and termination of plant rejuvenation are regulated by complex regulatory pathways and multiple levels of cellular machinery^[16]^. After rejuvenation, clear phenotypic and physiological changes can usually be observed. For example, Ananieva et al.^[20]^ found that declines in net photosynthetic rate and primary photochemical activity of PSII in senescing cotyledons of *Cucurbita pepo* were reversed by removal of the upper epicotyl.

Several studies on annual and perennial plants have demonstrated that microRNAs and transcription factors play critical regulatory roles in the processes of plant maturation and rejuvenation^[14,23,24]^. miR156 has been shown to prolong the expression of juvenile traits, its expression levels are higher in juvenile plants, whereas those of miR172 are higher during maturation^[24−27]^. Many genes have been found to function in the vegetative phase change, such as *SQUAMOSA PROMOTER-BINDING PROTEIN-LIKE* (SPL)^[25]^, *SUPPRESSOR of OVEREXPRESSION of CONSTANS 1* (SOC1)^[26]^, DAL1(a MADS-box gene)^[27,28]^, and *APETALA2-LIKE* gene^[17,29]^. The miR156 and miR172 gene families are strongly conserved and are involved in mediating vegetative phase changes in a larger number of plants^[30]^. The target gene of miR156, SPL, acts in multiple pathways including regulating changes in flowering time and vegetative phase changes, and the target gene SPL9 acts as a bridge to enable miR156 to regulate the expression of miR172^[31]^. These studies have revealed some of the molecular mechanisms that underlie vegetative phase changes and have identified candidate genes that can serve as molecular markers of these changes. Although numerous juvenile clones have been obtained through vegetative propagation in black locust, few of the molecular markers that regulate rejuvenation during vegetative propagation have been identified. Although different individuals from the same ortet are relatively consistent, plants produced by different methods of propagation vary in growth, development, and time to maturity. Juvenile characteristics and physiological differences associated with cyclophysis and topophysis in the aboveground parts of trees have been shown to greatly increase the efficiency of vegetative propagation and the renewal rate of aging individuals^[8,32,33]^. However, different rejuvenation methods have not previously been compared in black locust, nor has plantlets development been evaluated potential as a function of vegetative propagation methods. The best process for regeneration of plantlets from different vegetative organs remains unclear. Without an understanding of this process, there is a lead to the risk that individuals created using certain rejuvenation methods will undergo premature aging during long-term growth.

In this study, we evaluated the effects of three vegetative propagation methods (shoot cutting, root sprouting, and root cutting) on the rejuvenation of *R. pseudoacacia*, using seedling reproduction as a control. We also analyzed age-related small RNAs, genes, and transcription factors to determine whether they could be used as molecular markers for rejuvenation of black locust trees. Our goals were to make better use of black locust rejuvenation characteristics, to design an optimal strategy for plant regeneration, and to use this strategy for vegetative propagation of black locust.

## MATERIALS AND METHODS

### Plant materials and propagation methods

Fifteen mature wild *R. pseudoacacia* trees were selected as mother trees (MTs) and their ages were determined by counting tree ring numbers using an increment borer at breast height (1.3 m). Three types of plantlets (shoot-cutting plantlets [SCs], root-cutting plantlets [RCs], and root-sprouting plantlets [RSs]), were created from current-year branches and roots of MTs, and seed-derived seedlings (SSs) were obtained from semi-sib seeds of each mother tree. The experiment was performed at Lvcun Forest Farm in Luoyang, Henan Province, China (34°22'40.8" N, 111°19'48.6" E) in June 2020. The annual average temperature was 13.7 °C, and the average annual precipitation was 606 mm.

SSs were cultured in a 2:2:1 volumetric ratio of vermiculite, perlite, and peat, with an air-filled porosity of 42%. Seed pretreatment was required before sowing to break exogenous dormancy, as the hard black locust seed coat is impermeable to water and gases, preventing them from reaching the embryo. Seeds were pre-treated by soaking in water and then exposing them to different temperatures for different amounts of time (75 °C, 16 h; 85 °C, 24 h; 95 °C, 8 h). Seeds were sown after the germination treatment, and the seed-derived seedlings were transplanted to the field.

Healthy, semi-lignified shoot cuttings (~10 cm in length) from current-year MTs branches were selected from the lower part of the crown, and leaves and buds were removed from the lower part of each shoot cutting. The cutting base was dipped in a rooting induction solution of 1,500 mg/L 3-indolebutyric acid (IBA) for 5 s and then planted in medium consisting of vermiculite, perlite, and peat in a 2:2:1 volumetric ratio. The cuttings were exposed to natural sunlight, and the temperature of the rooting medium was maintained at 23 ± 5 °C to promote growth of the SCs. The resulting plantlets were transplanted to the field^[34]^.

RCs were obtained from root cuttings of the MTs (~3–5 cm diameter, ~10–20 cm length). The cuttings were placed horizontally with a spacing of 10–20 cm and covered with loose medium consisting of vermiculite, perlite, and peat in a 2:2:1 volumetric ratio. The resulting plantlets were transplanted to the field.

To produce RSs, woody roots of selected MTs were excavated to promote treatment at the base of the trunk at 50 cm intervals to the end. Promoted positions were cleaned and naturally dried, then girdled around ¼ of its circumference with a root cutter. A hormone solution was prepared by dissolving 100 mg/L 1-naphthaleneacetic acid (NAA), 90 mg/L indole acetic acid (IAA), and 300 mg/L gibberellin (GA3) in lanolin and was then applied to the cut site.

### Measurement of growth traits

In mid-September 2020, all characteristics of the plantlets were measured using a tape measure with an accuracy of 1 mm and a Vernier caliper with a precision of 0.1 mm for height and basal diameters. The leaf areas of fresh leaves were measured using an image scanner (Epson V39, Epson, China) and Fiji Image J software (ImageJ 2.1.0; Java 1.8.0-172).

### Morphological and anatomical observations

Shoot tips (~0.5 cm in length) were cut from the tops of current-year branches and fixed in formaldehyde-acetic acid-ethanol fixative. Paraffin sectioning and observation were performed as described previously.^[35]^ Anatomical structures of the shoot tips were observed using an inverted microscope (Leica DMI4000B, Germany), and development of shoot tip cells and tissues were analyzed statistically. Chloroplast grana lamellae, chloroplast membranes, mitochondria, and starch grains in the fourth pair of compound leaves from the plantlets and MTs were investigated by Qingdao Science Innovation Quality Testing Co. (Qingdao, China) using transmission electron microscopy (TEM). Three independent replicates were observed for each plantlet sample.

### Physiological and photosynthetic characteristics

We evaluated differences in photosynthetic activity of plantlets and seedlings obtained using different methods. Leaves of plantlets, seedlings and MTs were analyzed using an LI-6400 portable photosynthesis system (LI-6400C, LI-COR, USA) in mid-September 2020; measurements were made in the middle of each leaf, avoiding the leaf veins. For the RS samples, plantlets for photosynthetic measurements were selected from the periphery of the mother tree crown, avoiding the influence of the mother tree on plantlet light interception whenever possible.

For physiological measurements, fresh leaf samples (the third pair of leaflets from the tip of healthy compound leaves) were harvested from MTs, plantlets, and seedlings; the samples were wrapped in aluminum foil, immediately frozen in liquid nitrogen, and stored at −80 °C for letter experimentation. RSs were selected from the periphery of MTs whenever possible to reduce influence of MTs. The amounts of total protein, soluble saccharides, malondialdehyde (MDA), and total phenols and the activity of superoxide dismutase (SOD) and L-phenylalanine ammonia-lyase (PAL) were measured using enzyme activity kits (A045, A145, A003, A001, A137, A143; JianCheng Bioengineering, Nanjing, China). At least three independent replicates were performed for each sample.

### RNA extraction and gene expression analysis

To analyze the expression of small RNAs and genes (including those encoding transcription factors) (Table 1) in plantlets, seedlings and MTs, total RNA was extracted from replicate leaf samples using a HiPure Universal miRNA Kit (R4310, Magen Biotechnology, Beijing, China) according to the manufacturer’s instructions. The OD260/280 ratio for all RNA samples was determined using a NanoDrop2000 spectrophotometer (Thermo Scientific, Waltham, MA, USA), and the integrity of the RNA samples was verified by agarose gel electrophoresis. Small RNA reverse transcription was performed to complete the cDNA synthesis reaction (stem-loop-specific) using the Mir-X miRNA First-Strand Synthesis Kit (638313, Takara Bio, Beijing, China) according to the manufacturer’s instructions. The transcript levels of miR156 and miR172 were quantified using a Mir-X miRNA RT-PCR TB Green Kit (639676, Takara Bio, Beijing, China). Gene expression was analyzed by qRT–PCR using a PrimeScript RT Master Mix (RR036A, Takara Bio, Beijing, China). All reactions were performed on the Applied Biosystems 7500 Fast Real-Time PCR System (Thermo Fisher, USA). Expression levels were calculated by the 2^−ΔΔCᴛ^ method. PCR primers used to analyze the expression of candidate genes were designed using Primer3 (www.ncbi.nlm.nih.gov/tools/primer-blast/) and are listed in Supplemental Tables S1 & S3.

**Table 1:** Key miRNAs and genes known to function in plant vegetative phase change.

miRNA/Gene	Species	Juvenile	Adult	Function	References
miR156	*A. thaliana*	Highly expressed	−	Prolongs juvenility and delays the onset of the adult phase.	[30,36]
miR172	*A. thaliana*	−	Highly expressed	Its expression is regulated by miR156 via SPL, thereby regulating plant vegetative phase change.	[36,37]
SPL3/4/5	*A. thaliana*	−	Highly expressed	Regulates flowering time and phase change	[38,39]
SPL9/15	*A. thaliana*	−	Highly expressed	Promotes juvenile-to-adult phase transition and flowering; directly regulates genes involved in trichome formation.	[36,40]
SPL2/10/11	*A. thaliana*	−	Highly expressed	Regulates leaf shape and trichome distribution with shoot maturation in the reproductive phase.	[36,39]
SPL6/13	*A. thaliana*	−	Highly expressed	Regulates the switch from cotyledon to vegetative leaf stage.	[36]
TOE1/2	*A. thaliana*	Highly expressed	−	Represses flowering	[39]
*VAL*1/2	*A. thaliana*	−	Highly expressed	Both* VAL1* and *VAL2* regulate vegetative phase change by constitutively decreasing miR156 levels and indirectly promoting SPL expression	[27]
SPL, *SQUAMOSA PROMOTER BINDINGPROTEIN-LIKE*; TOE, *TARGET OF EAT*; *VAL*, *VIVIPAROUS/ABI3-LIKE.*					

### Statistical analysis

Data were analyzed using analysis of variance followed by Tukey's honestly significant difference (HSD) mean separation procedure with IBM SPSS Statistics 26.0 (IBM Corp, Armonk, NY, USA). Means were considered to be significantly different when P values were less than 0.05 according to an ANOVA F-test. The results are presented as the mean ± SE (standard error).

## RESULTS

### Rejuvenated plantlets obtained by different methods

**Figure 1:**
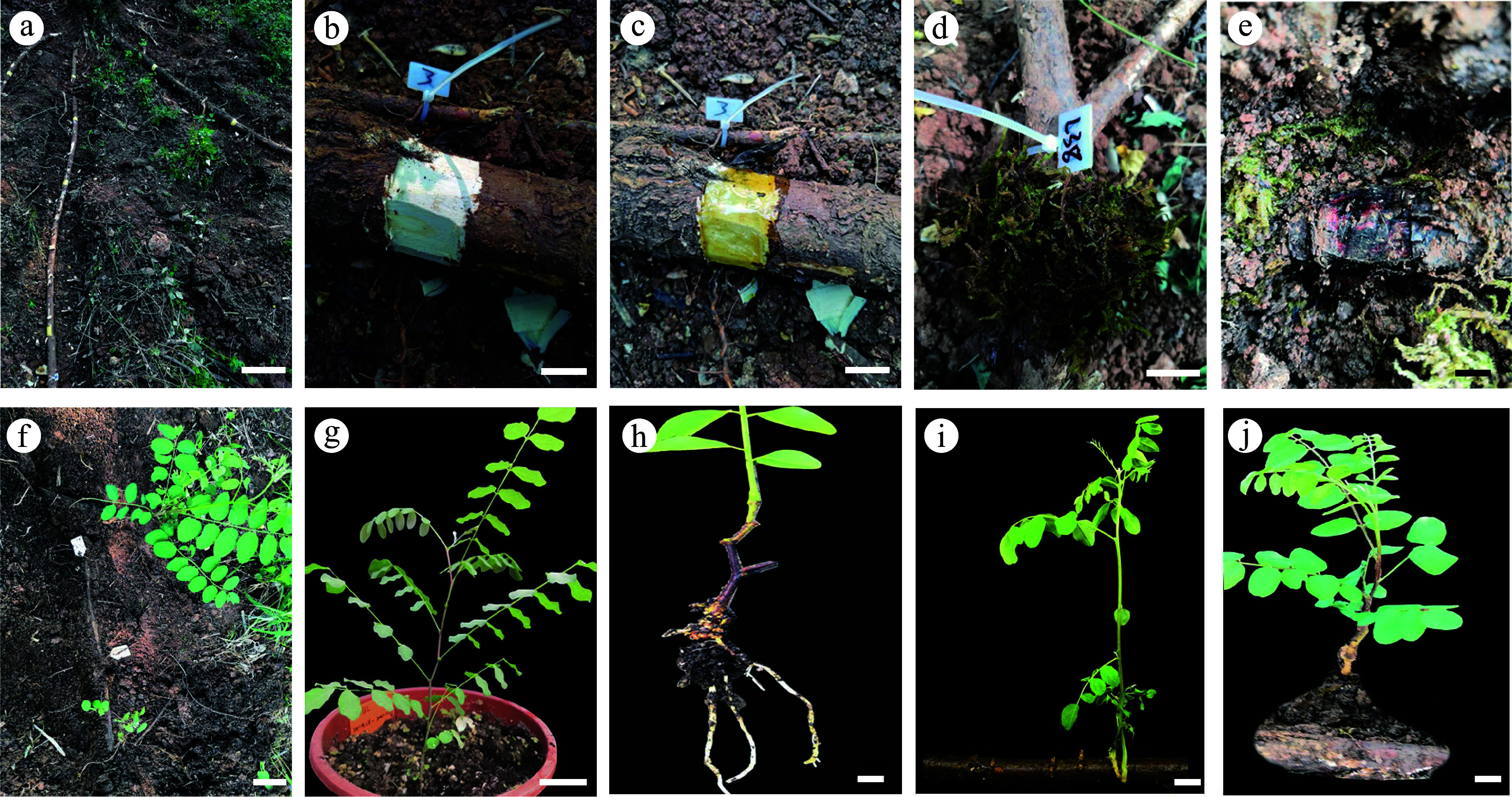
Propagation by root-sprouting and examples of black locust plants produced with different propagation methods. (a) Exposure of a woody root. (b) Girdling one quarter of the root circumference. (c) Hormone treatment. (d) Moisturizing treatment. (e) Treated root after 15 d. (f) Germinated plantlets in root-sprouting treatment. (g) Seed-derived seedlings. (h) Plantlets obtained by root-sprouting. (i) Plantlets obtained from root-cuttings. (j) Plantlets obtained from shoot-cutting. Scale bars: (a) 50 cm, (b)−(f) 2 cm, (g), 5 cm, (h)−(j) 2 cm.

Fifteen adult black locust mother trees (MTs) were divided into three groups (MT1-MT5, MT6-MT10, and MT11-MT15) for experiments and analysis. RSs were obtained by scraping the roots of each MTs *in situ*, RCs were obtained from root cuttings of the same MT *in vitro*, and SCs were obtained from shoot cuttings of the same MT *in vitro*. Seeds from the same MT were sown to obtain the sexually propagated control seedlings (i.e., SSs). Examples of vegetatively propagated plantlets and sexually propagated seedlings are shown in Fig. 1. The numbers of SSs, RSs, RCs, and SCs obtained from each of the three MT groups are shown in Table 2.

**Table 2:** Ages of mother trees and numbers of seedlings and plantlets obtained from each mother tree.

MT	MT Age (y)	SS (N)	SC (N)	RC (N)	RS (N)
MT1	18	15	8	7	14
MT2	19	15	1	5	7
MT3	21	15	3	10	13
MT4	17	15	3	0	16
MT5	24	15	8	6	21
**Total**		75	23	29	71
MT6	16	15	2	0	18
MT7	19	15	2	0	17
MT8	17	15	8	5	10
MT9	22	15	4	10	8
MT10	18	15	4	0	12
**Total**		75	20	15	65
MT11	20	15	13	0	7
MT12	19	15	6	6	18
MT13	22	15	5	3	9
MT14	24	15	14	5	5
MT15	17	15	6	1	12
**Total**		75	44	15	51
**Grand total**		225	87	59	187
MT, mother tree; SS, seed-derived seedling; SC, shoot-cutting plantlet; RC, root-cutting plantlet; RS, root-sprouting plantlet.					

### Growth and leaf traits of plantlets, seedlings and mother trees

**Figure 2:**
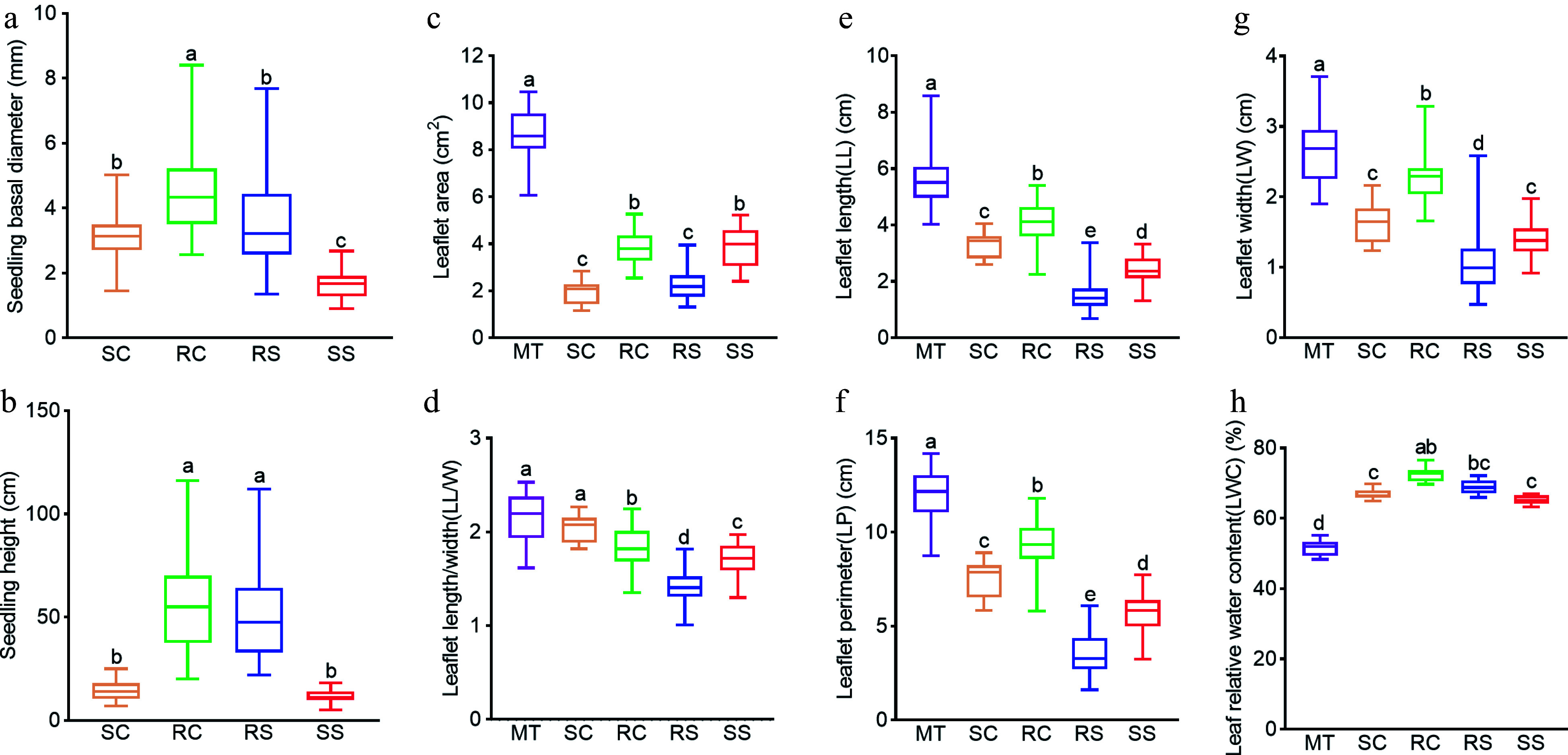
Growth and leaf traits of plantlets, seedlings and mother trees. (a) Basal diameter. (b) Seedling height. (c) Leaflet area. (d) Leaflet length/width. (e) Leaflet length. (f) Leaflet perimeter. (g) Leaflet width. (h) Leaf relative water content. Different lowercase letters above the bars indicate significant differences between plant materials (*p* < 0.05, ANOVA and Tukey's honestly significant difference test). Abbreviations: SS, seed-derived seedlings; RS, root-sprouting plantlets, RC, root-cutting plantlets; SC, shoot-cutting plantlets; MT, mother trees.

During vegetative growth, the vegetative phase change is accompanied by changes in leaf size and shape. We therefore measured leaf traits of seedlings and vegetative plantlets, using leaves of MTs as a control. We also compared the differences of growth traits between seedlings and vegetative plantlets. The basal diameters of SCs, RCs, and RSs were significantly higher than those of SSs (Fig. 2a). The height of RCs and RSs was significantly greater than that of SCs and SSs, but there were no significant differences between the heights of SCs and SSs or between the heights of RCs and RSs (Fig. 2b).

There were significant differences in leaf traits between MTs and plantlets/seedlings. In almost all cases, leaflet area, leaflet width, leaflet length, leaflet length/width ratio, and leaflet perimeter were significantly lower in plantlets/seedlings than in MTs (Fig. 2c, e-g). Among the plantlets, RCs had significantly larger values of almost all leaf traits. By contrast, the leaf relative water content was significantly higher in plantlets and seedlings than in MTs (Fig. 2g). Although growth and leaf traits between seedlings and vegetative plantlets could not be used as a good criterion, it also showed that root-propagated plantlets outperformed shoot-cutting plantlets in growth and development in black locust.

### Leaf photosynthesis of plantlets, seedlings and mother trees

The instantaneous leaf photosynthetic rate differed significantly between plantlets/seedlings and MTs. Among the plantlets, RSs had significantly lower photosynthetic rates than SSs, SCs, and RCs, but photosynthetic rates did not differ significantly among SSs, SCs, and RCs (Supplemental Fig. S1a). Stomatal conductance and transpiration parameters showed a pattern similar to that of photosynthetic rate between plantlets, seedlings and MTs (Supplemental Fig. S1b, S1d). The intercellular CO2 concentration was significantly lower in RCs than in SSs, SCs, RSs, and MTs (Supplemental Fig. S1c).

### Physiological traits of plantlets, seedlings and mother trees

**Figure 3:**
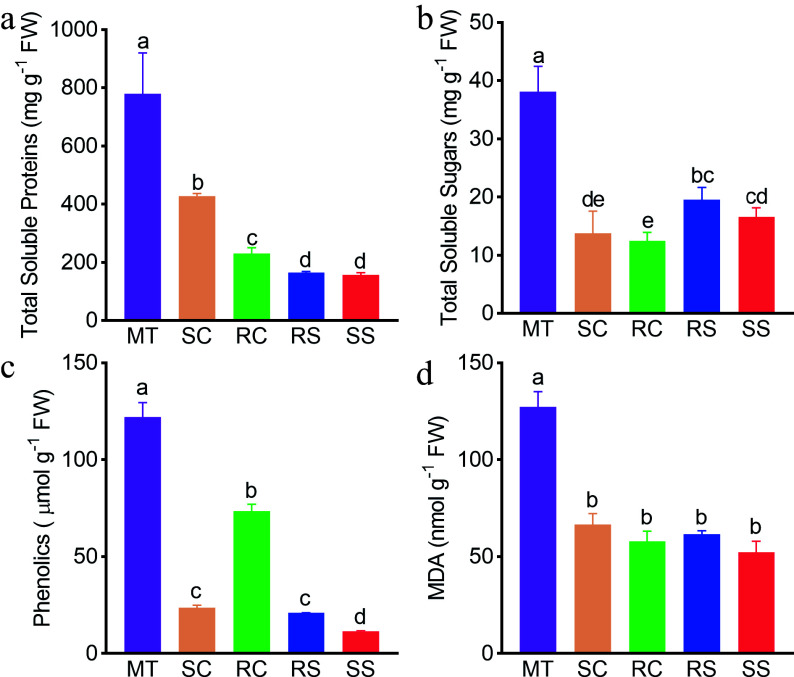
Physiological traits of juvenile plantlets/seedlings and mother trees. (a) Total soluble protein. (b) Total soluble sugars. (c) Phenolic content. (d) MDA content. Different lowercase letters above the bars indicate significant differences among plant materials. Abbreviations: SS, seed-derived seedlings; RS, root-sprout plantlets; RC, root-cutting plantlets; SC, shoot-cutting plantlets; MT, mother trees. MDA, malondialdehyde.

The amount of total protein was significantly higher in MTs than in plantlets/seedlings, and SCs had the highest total protein levels among the plantlets/seedling types (Fig. 3a). There were no significant differences in MDA and phenolic content among the plantlets/seedlings, with the exception of RCs, which had a higher phenolic content (Fig. 3d). Total soluble sugars and total phenolic content were significantly higher in MTs than in plantlets/seedlings (Fig. 3b, c), but SOD contents did not differ between these plant materials (Supplemental Fig. S1e). Total protein, total soluble sugars, total phenolics, MDA contents, and PAL activity were significantly higher in biennial RSs than in biennial RCs (Supplemental Fig. S2). These results demonstrate that a number of key metabolites and enzymes were present at higher levels in MTs than in the younger plants, and SSs were the youngest with regard to the pattern of change in physiological traits.

### Effect of rejuvenation methods on stem anatomy

**Figure 4:**
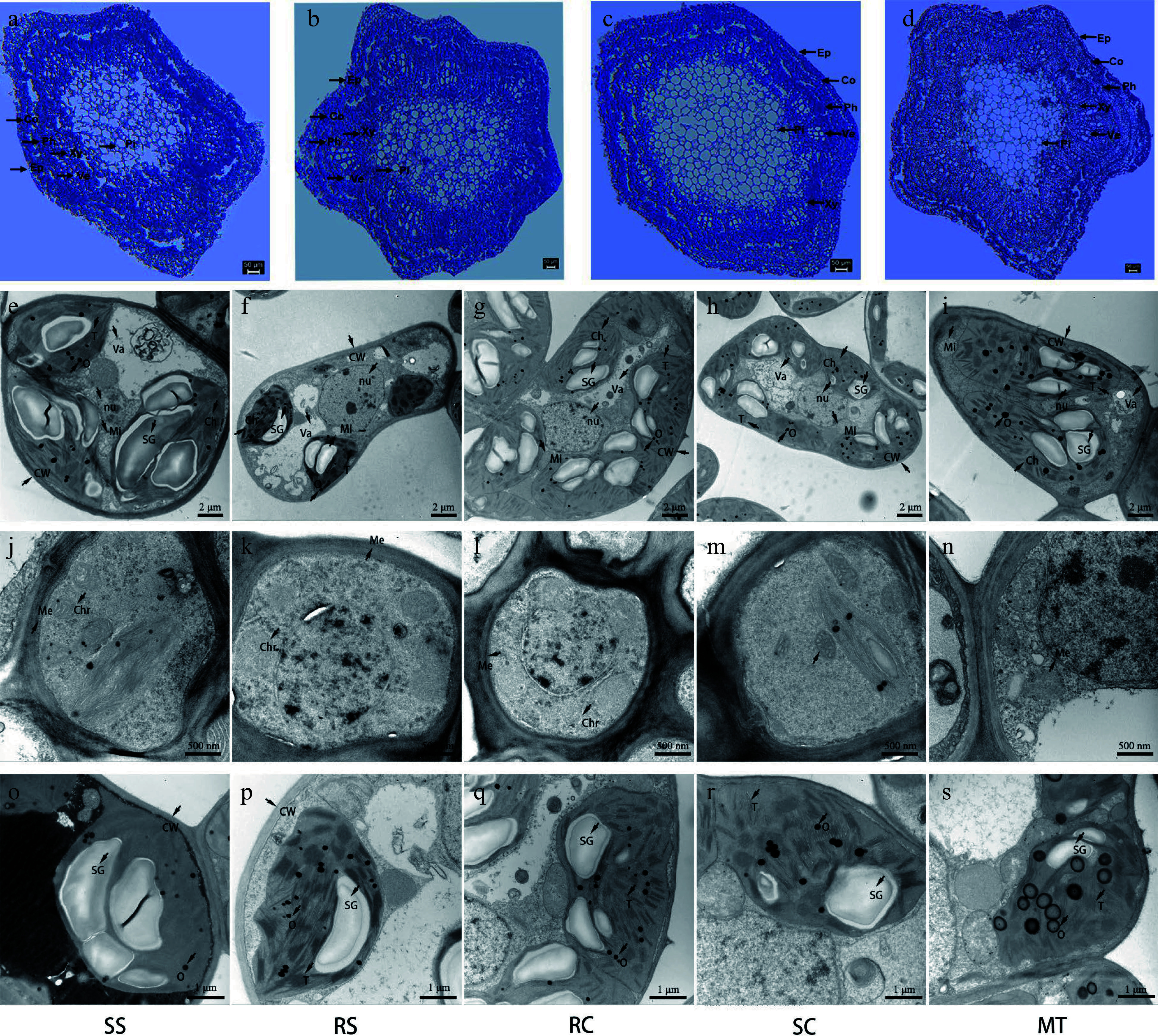
Shoot cross-sectional anatomy and leaf-cell ultrastructure of plantlets. (a)–(d) Stem cross sections of (a) SS, (b) RC, (c) RS, and (d) SS. (e)–(s) Ultrathin sections of leaves. SS, seed-derived seedlings; RS, root-sprout plantlets; RC, root-cutting plantlets; SC, shoot-cutting plantlets; MT, mother trees. Abbreviations: Ep, Epidermis; Co, Cortex; Ph, Phloem; Xy, Xylem; Ve, Vessel; Pi, Pith; CW, cell wall; Ch, chloroplast; Mi, mitochondrion; nu, nucleus; SG, starch granule; Va, vacuole; T; thylakoid; O, osmiophilic droplet; Me: nuclear membrane; Chr, chromatin. Scale bars: (a), (b) 50 µm, (e)−(i) 2 µm, (j)−(n) 500 nm, (o)−(s) 1 µm.

The shoot tip is an important vegetative organ, and we therefore examined anatomical differences in the shoot tips of SSs, SCs, RCs, and RSs. The phloem is mainly composed of parenchymal cells, which were loosely arranged in SSs, RCs, and RSs but tightly arranged in SCs (Fig. 4a–d). The vessels were nearly round, oval, or irregularly polygonal in shape. Relative to the other plant types, the SSs had less xylem, which was more loosely arranged and showed substantially less lignification. The degree of lignification in RCs and RSs was similar. Xylem of the SCs showed the tightest organization and greatest lignification of all the plant types.

Pith cells were tightly and irregularly distributed in the sexually-propagated SSs compared with the vegetatively propagated SCs, RCs, and RSs. The pith ratio was higher in biennial RSs than in RCs, and there were no significant differences in other structures (Supplemental Fig. S3). In conclusion, the shoot structure of the Scs had the most mature appearance, whereas that of the germinated seedlings had the most juvenile appearance.

### Leaf-cell ultrastructure of plantlets, seedlings and mother trees

During plant development, changes in foliar organs can reflect changes in the age of individuals. In our study, the vacuole was more easily observed (Fig. 4e−i), the leaf cell walls were thinner, and the volume ratios of mesophyll and chloroplasts per unit area was larger in plantlets/seedlings than in MTs (Fig. 4e−i). The volume ratios of mesophyll, chloroplasts, and mitochondria were also significantly larger in SSs than in the vegetatively-derived plantlets (Fig. 4e), and there were no significant differences in these ratios between RSs and RCs. The volume ratios of mesophyll, chloroplasts, and mitochondria were significantly lower in SCs (Fig. 4h). The nuclei maintained intact nuclear membranes and nucleoli in leaf cells of plantlets/seedlings, and the nucleoli disappeared (Fig. 4j−m). The chromatin progressively condensed into clumps inside the nucleoplasm of MTs (Fig. 4n). The depletion of cytoplasm during plant development, accompanied by organelle and chloroplast degradation, are the first signs of visible damage^[41]^. The number of vesicles originating from chloroplast degeneration was significantly greater in the leaf of MTs compared with those of plantlets/seedlings (Fig. 4s), and few vesicles were observed in the SCs (Fig 4r). Vesicles were difficult to see in SSs, RCs, and RSs (Fig. 4o−q). The number of osmiophilic droplets was significantly lower in plantlets/seedlings than in MTs (Fig. 4e−i), but the size of these droplets was significantly larger in plantlets (Fig. 4o−s). In leaf sections from biennial RSs and RCs, there were no significant differences in the number of osmiophilic droplets, but there were significantly more osmiophilic droplets in biennial RSs and RCs than in annual RSs and RCs (Supplemental Fig. S4).

### Gene expression in plantlets, seedlings and mother trees

**Figure 5:**
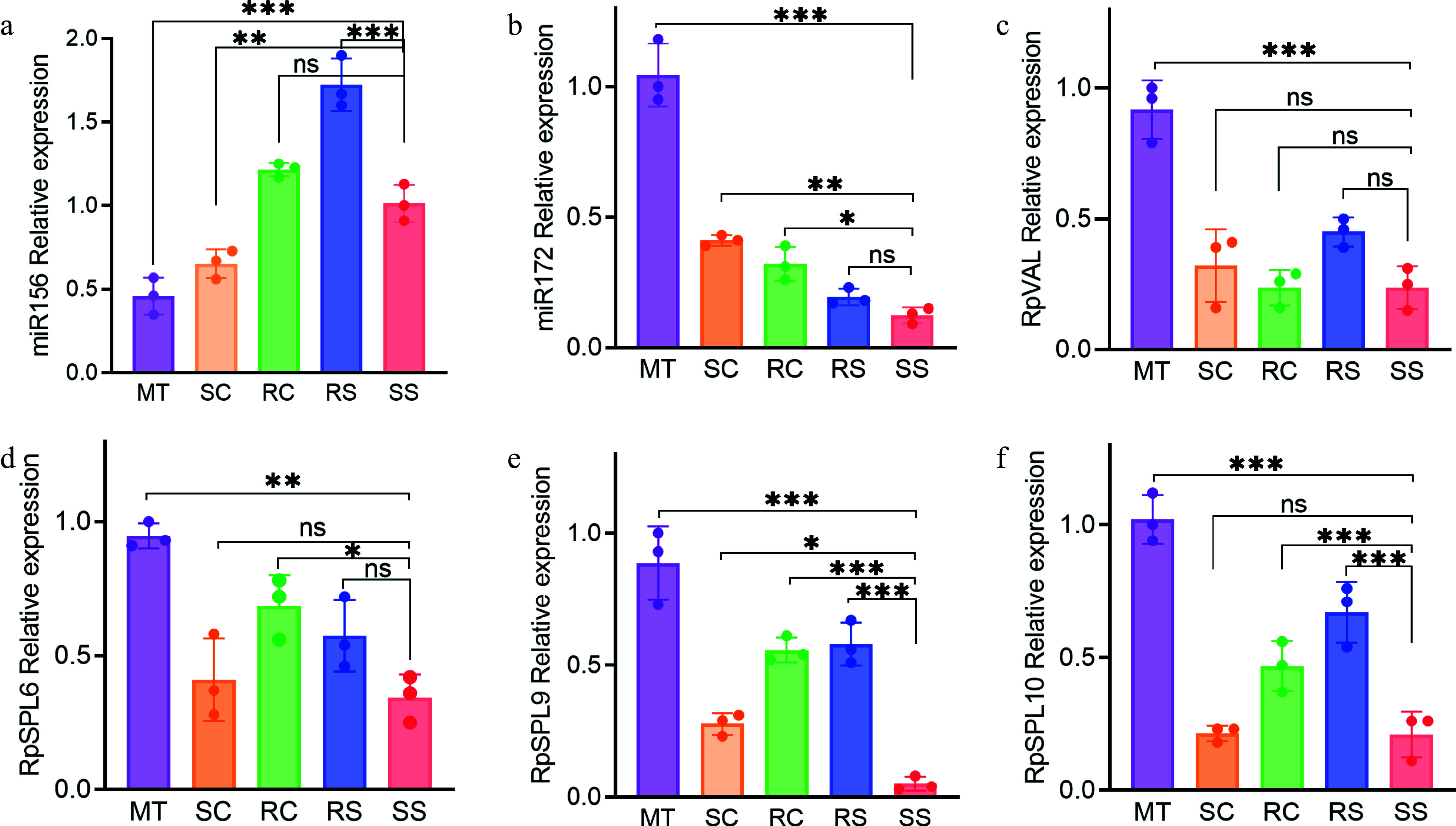
Relative expression of genes and microRNAs in the plantlets/seedlings and mother trees. (a) miR156, (b) miR172, (c) *RpVAL*, (d) *RpSPL6*, (e) *RpSPL9*, and (f) *RpSPL10*. Asterisks indicate statistically significant differences compared to the seed-derived seedlings (* *p* < 0.05; ** *p* < 0.01; *** *p* < 0.001; ns, no significant difference). Abbreviations: SS, seed-derived seedlings; RS, root-sprout plantlets; RC, root-cutting plantlets; SC, shoot-cutting plantlets; MT, mother trees.

With increasing attention being paid to plant rejuvenation, rejuvenation-associated genes (RAGs) and rejuvenation-associated small RNAs (RA-sRNAs) and their expression patterns have been studied extensively in leaves^[16]^. Here miR156 expression levels were significantly higher in SCs (1.46×), RCs (2.6×), RSs (3.7×), and SSs (2.17×) than in MTs (Fig. 5a), and miR172 expression showed the opposite pattern (Fig. 5b). *VAL* genes promote the vegetative phase transition by lowering the overall level of miR156 expression^[42]^. Consistent with the miR156 results, *VAL1* gene expression was significantly lower in plantlets/seedlings than in MTs, although there were no significant differences in *VAL1* expression among the plantlets/seedlings (Fig. 5c). The expression of several miR156 target genes, including *SPL6*, *SPL9*, and *SPL10*, was also significantly lower in plantlets/seedlings than in MTs (Fig. 5d−f). Collectively, these results demonstrate differences in the expression of some RAGs and RA-sRNAs among plantlets seedlings, and MTs.

## DISCUSSION

The use of vegetative propagation in forestry has a long history, enabling the preservation of superior genotypes and desirable traits for use in clonal forestry^[43]^. However, clonal plants propagated from different plant parts (root sprouts, stem scions, twigs, etc.) often retain some of the 'growth habits' of the propagules collected from the mother tree^[8]^, and development of methods for rejuvenation of vegetatively propagated plants is thus important for clonal forestry. Rejuvenation can restore the growth vigor and development potential of plants propagated from aged trees and studies on programmed plant growth contribute to our understanding of plant rejuvenation^[44]^. In previous studies, researchers have examined the cytological and ultrastructural changes in plant epidermal cells, as well as the changes in physiology and gene expression, associated with rejuvenation^[20,45−47]^. Although numerous juvenile clones have been obtained though vegetative propagation, the development and selection of appropriate propagation methods is important for efficient rejuvenation of *R. pseudoacacia.*

The vegetative phase change has been studied in many plant species, and the existence of this phenomenon and its significance for plant development are gaining greater appreciation^[11,48]^. However, as the reverse process of the phase changes, there is a lack of data on the juvenile status of plantlets created through different propagation methods. Here, we analyzed differences in growth rate, developmental morphology, and physiology of plantlets/seedlings obtained using different propagation methods. Some of the mature black locust trees produced only a small number of plantlets by root cutting. By contrast, root sprouting produced larger numbers of plantlets perhaps because of nutrition provided by the mother trees and/or the provided by the mother trees hormones during the propagation process.

Many plants show little variation in phenotypic features during shoot plant development, making the onset of the vegetative phase change difficult to observe. In this study, we found that the leaf length/width ratio and leaf area were lower in juvenile black locust trees than in their mature MTs. These results are consistent with the findings in *Olea europaea* that leaves of juvenile trees are smaller and rounder than those of adult trees^[49]^. The vegetative phase change may involve traits unique to a particular species, and differences in leaf morphology may represent such a trait in black locust, a finding that could be useful for future research on this species and its propagation.

Previous studies have showed that recovery of the photosynthetic rate after rejuvenation is associated with larger chloroplasts in the plantlet leaves^[22]^. Here, we found higher leaf photosynthetic rate in plantlets/seedlings than in MTs (Supplemental Fig. S1). Photosynthetic rates were lower in RSs than in other plantlets/seedlings, perhaps because their light interception was limited by the presence of the MTs^[10]^. In woody plants, changes in esterase and peroxidase isozymes are important markers of maturity, differentiating mature trees from juvenile^[16]^. Our peroxidase isozyme and sugar results were consistent with the results of other studies^[20]^. It was noted that the enzymatic activity and amounts of sugar and total soluble protein were significantly higher in plantlets after rejuvenation by sexual and asexual propagation than in mature trees. However, no significantly consistent patterns of change among annual plantlets were observed in this study (Fig. 3).

The extent to which plants can re-establish their juvenile status is still unknown. Changes in plant morphological characteristics are evident with age and reflect different plant developmental states^[50]^. In particular, the phase transition from juvenile to adult is accompanied by gradual changes in leaf morphology and anatomy^[21,51]^. As a process of return to the juvenile state from the adult state, rejuvenation is also accompanied by morphological changes, but the renewal of juvenile features may not occur consistently and simultaneously. Leaf development is associated with major changes in leaf ultrastructure, including changes in the chloroplast, vacuole, and nucleus. In particular, there is a strong contrast between the vacuole characteristics of adult and juvenile leaves. Also, the periderm is thicker and lignification is greater in older trees of *Populus alba*^[52]^. Our results showed that SCs had a high degree of lignification, suggesting that the juvenile status of SCs may be less established than that of RCs, RSs, and SSs. In the juvenile-to-adult transition of Mediterranean pines, the leaf dry mass per unit area and cell wall thickness of young individuals increased, the mesophyll and chloroplast volume ratios decreased, and the photosynthetic activity declined^[51]^. In *Beta vulgaris*, starch granules decreased in size in the later stages of leaf senescence^[53]^. In our study, starch grain sizes decreased and lipid droplets appeared in leaf cells of SSs, RSs, RCs, SCs, and MTs (Fig. 4e−i). The number and volume of starch granules in leaf cells decreased, whereas the number and volume of osmiophilic droplets increased. This suggests that the leaves of SCs had an older developmental status. These results indicate that the root possesses embryonic cell characteristics, and the farther the aboveground portion of the tree is from the root, the older the tree^[54]^. This observation raises the possibility that rejuvenation characteristics associated with asexual propagation from roots may be consistent with those of sexual propagation from seeds.

Phenotypic changes are genetically regulated by endogenous factors in plants^[55]^. The expression levels of miR156 declines with plant age, whereas *SPL* and miR172 expression levels increase^[30, 56]^. However, it’s not clear whether these results are limited to *Populus* or other model tree species – or whether they are generally accepted for multiple plant species. We therefore analyzed expression patterns of age-related small RNA, genes, and transcription factors in this study. Our results suggest that miR156 and SPLs may play roles in the regeneration and/or rejuvenation of black locust plantlets, similar to their roles in the normal vegetative phase change of black locust. However, their roles in black locust rejuvenation require additional research.

## CONCLUSIONS

We showed that most characteristics of plantlets produced by root sprouting were similar to those of seed-derived seedlings, including leaf traits and leaf anatomical structures. However, there were significant differences between plantlets derived from shoot cuttings and the seed-derived seedlings. Our results also suggested that age-related small RNAs and transcription factor genes may function not only as the molecular markers for phase transitions, but also as potential juvenility markers in black locust. Root-based rejuvenation appeared to offer advantages for physiology, growth, and development compared with rejuvenation from shoot cuttings. Our results provide useful information for the optimal propagation of woody trees and for further research into the mechanisms of regeneration from roots.

## SUPPLEMENTARY DATA

Supplementary data to this article can be found online.
