# Supplementary material for: Effects of the vegetative propagation method on juvenility in Robinia pseudoacacia L
Source: For Res (Fayettev). 2022 Dec 5;2:17. doi: 10.48130/FR-2022-0017 (PMC11524284; doi:10.48130/FR-2022-0017)
Supplement: Supplementary file 1 — Supplementary data to this article can be found online. [file FR-2022-0017-S1.zip › 10.48130_FR-2022-0017-Suppl-FigureS2.docx]

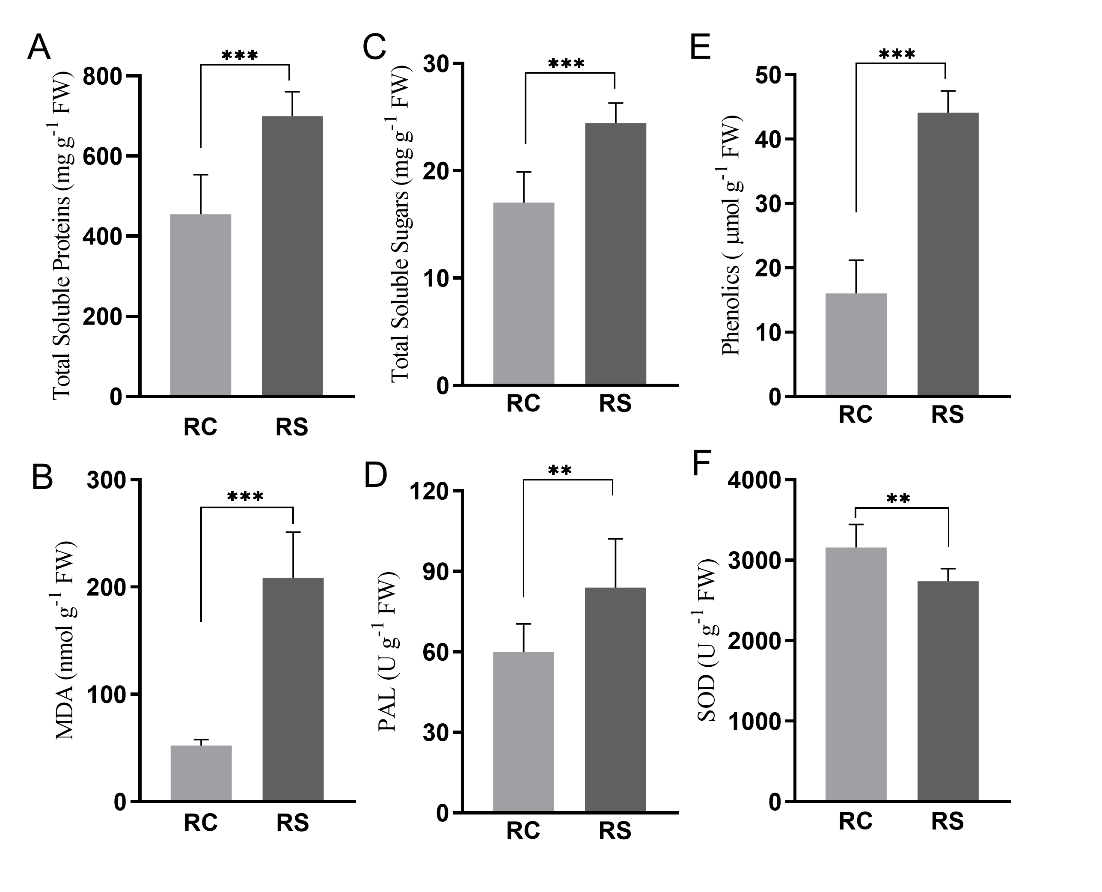


**Fig. S2.** Physiological traits of biennial plantlets. (A)Total soluble protein, (B)Total soluble sugars, (C)Phenolics, (D) MDA, (E) PAL, (F) SOD. Different lowercase letters above the bars indicate significant differences among the plant materials. Abbreviations: SS, seed-derived seedlings; RS, root-sprout plantlets, RC, root-cutting plantlets; SC, shoot-cutting plantlets; MT, mother trees; MDA, malondialdehyde; SOD, superoxide dismutase; PAL, L-phenylalanine ammonia-lyase.
