# Supplementary material for: Effects of the vegetative propagation method on juvenility in Robinia pseudoacacia L
Source: For Res (Fayettev). 2022 Dec 5;2:17. doi: 10.48130/FR-2022-0017 (PMC11524284; doi:10.48130/FR-2022-0017)
Supplement: Supplementary file 1 — Supplementary data to this article can be found online. [file FR-2022-0017-S1.zip › 10.48130_FR-2022-0017-Suppl-FigureS3.docx]

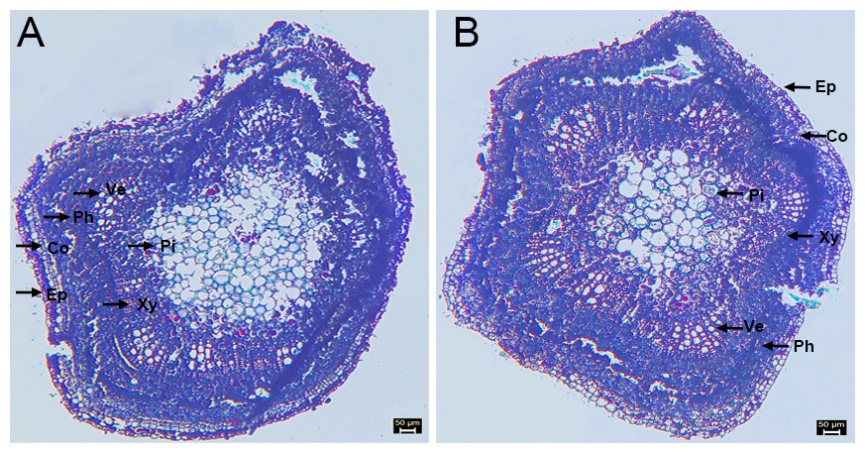


**Fig. S3.** Shoot cross-sectional anatomy of biennial plantlets. (A) root-sprout plantlets (RSs). (B) the RCs. Abbreviations: Ep, Epidermis; Co, Cortex; Ph, Phloem; Xy, Xylem; Ve, Vessel; Pi, Pith. Scale bars: 50µm.
