# Supplementary material for: Effects of the vegetative propagation method on juvenility in Robinia pseudoacacia L
Source: For Res (Fayettev). 2022 Dec 5;2:17. doi: 10.48130/FR-2022-0017 (PMC11524284; doi:10.48130/FR-2022-0017)
Supplement: Supplementary file 1 — Supplementary data to this article can be found online. [file FR-2022-0017-S1.zip › 10.48130_FR-2022-0017-Suppl-FigureS4.docx]

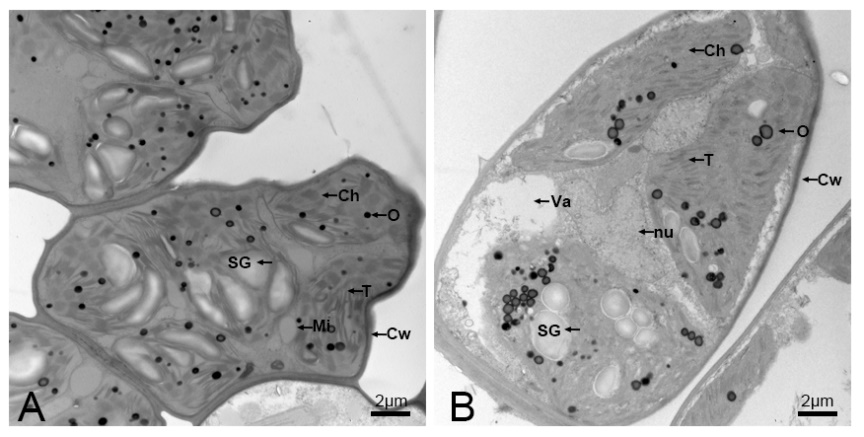


**Fig. S4.** Leaf-cell ultrastructure of biennial plantlets. (A) root-sprout plantlets (RSs). (B) root-cutting plantlets (RCs); Abbreviations: CW, cell wall; Ch, chloroplast; Mi, mitochondrion; nu, nucleus; SG, starch granule; Va, vacuole; T; thylakoid; O, osmiophilic droplet. Scale bars: 2µm.
