# Supplementary material for: Effects of the vegetative propagation method on juvenility in Robinia pseudoacacia L
Source: For Res (Fayettev). 2022 Dec 5;2:17. doi: 10.48130/FR-2022-0017 (PMC11524284; doi:10.48130/FR-2022-0017)
Supplement: Supplementary file 1 — Supplementary data to this article can be found online. [file FR-2022-0017-S1.zip › 10.48130_FR-2022-0017-Suppl-TableS1.docx]

| Supplementary Tables 1 RT-qPCR primers informations used in this study | | |
| --- | --- | --- |
| Reverse transcription | Primer name | Sequencing information (5’-3’) |
|  | miR156-T | GTCGTATCCAGTGCAGGGTCCGAGGTATTCGCACTGGATACGACACGTGA |
|  | miR172-T | GTCGTATCCAGTGCAGGGTCCGAGGTATTCGCACTGGATACGAC |
|  | U6-T | GTGCAGGGTCCGAGGTTTTGGACCATTTCTCGAT |
| RT qPCR | RpACT-F | CGCTTGCCTTGGATTATGAACA |
|  | RpACT-R | CGGATGGCTGGAACAGAACTT |
|  | RpU6-F | GGAACGATACAGAGAAGATTAGCA |
|  | RpU6-R | GTGCAGGGTCCGAGGT |
|  | miR156-F | CCTATCTCTGCCTGCTTGACCT |
|  | miR172-F | CCGCCCGAATCTTGATGATG |
|  | miRNA-R | CAGTGCAGGGTCCGAGGTAT |
|  | RpSPL6F | GTGATGTTCACACCAAGACTGC |
|  | RpSPL6R | AAAGGGGTGTCAGAAACTTGGT |
|  | RpSPL9F | ACACCACACCAAGTGTTGAG |
|  | RpSPL9R | TGATGGATGGCTGCAACTTG |
|  | RpSPL10F | CTGGGATTCCTATGTCTCGTGG |
|  | RpSPL10R | AGACGAGGTAGTGGGATCTTCA |
|  | RpVAL1F | AGTGAATTCAGCCGCTGGAA |
|  | RpVAL1R | AATCTCCGGACAATCGCTCC |
