# Supplementary material for: Effects of the vegetative propagation method on juvenility in Robinia pseudoacacia L
Source: For Res (Fayettev). 2022 Dec 5;2:17. doi: 10.48130/FR-2022-0017 (PMC11524284; doi:10.48130/FR-2022-0017)
Supplement: Supplementary file 1 — Supplementary data to this article can be found online. [file FR-2022-0017-S1.zip › 10.48130_FR-2022-0017-Suppl-TableS2.docx]

The sequences of studied genes

RpSPL6 mRNA

ATGGAATCTTGGAGTTATGTCCCTGAAGAGAAAGGCTATTTGTTTTCTGATGAAATGGATTTTTCACTTGATGTTTTTATGAGAAGTAGAAAAGCATTGGTTGAATGGGACAACAAACGCTCATGTAGCTTTGAGAGAGATGAATTTAATTCAGACAAAGAAGTAGTTAAGAGCATGGAATTTGTGAACTTGGGATTTCCTGACTTGTTGGAAAAGTCTTTTCATGGTAGCCAACCTTTGGAGACATCAAGCTGTGATGTGGAAAGTAATTCTGGTAAAAGAGGGAATTCCTTCACCCATGTTATTGCTTTGGATTCATCTTTTGGGGAAGAAGAATCAGATTCAAAGCATTTAAGTTCTCTAATTGAATCCAAAACTCATGATTCCTCACTGATTGATTTGAAGCTAGGGAGATTAGCAGATTGTAAAGGTGCAAGCAGTGATAAAAATGCAAAAGAAAGCTTCACTTTAACATCTATACATCCAACCACGCTTACTAAGAGAGCTCGCACTTCCAGCTTACCTGCTCAAGCTCCTGTATGTCAAGTTTATGGTTGTAACATGGATCTTAGCTCCTCAAAAGATTACCACAAAAGGCATAAAGTTTGTGATGTTCACACCAAGACTGCTAAAGTTATTGTTAATGGCATTGAACAGAGGTTTTGTCAGCAGTGCAGCAGGTTCCATTTGCTAGCTGAGTTTGATGATGGTAAGCGAAGTTGTCGCAGGCGTCTAGCCGGACACAATGAACGCCGAAGGAAACCTCAGTTTGATTACATGACTGATAAACAGCACAAGATTCTTCAGTCATATCAAGGTACTAAGTATCTGGGGTCTTCATTACAGAACAGACCCCAGTTTGCCTTTCAAGATGTATTTCAAAGTGGCATCTTTTTCCCAGGAAAGCATGATCAAATCTATCAGAGTGGACATATCAAATTGGAAGAGGATTCAATTTGCAGTTCTCAACTAGCAGCACCTATCACACTTGGTCAGGAGTTATCCAGCTGTGCTCTCTCTCTTCTGTCAGATCAGTCACAGAATCCTTCACGCTTCTCACCACGAAATCCATTAGCTAGTTCCCTTGTCTTTCAGATGAATGACAGAGATGACCAAGTTTCTGACACCCCTTTGAGGATAAGCTCTATGGATAAATATGTACCAAATGAGTCCTTCCCATGTGGGATAAACTCTAATAAGGAAGTTATCAAGAATAGATCCACAACACTTTCTGATGCTGGTCATGCTCTTCAAGTCCACAGAGATGATATTTGCCAACCATCAGAATCATTCAATGCCACTGTTGATCTGTTTCAATTGTCTTCTCATCTTCAGAGGGTGGAGCAGCAAAGAAATTCTGTTTTGGTAAAGTGGGAAAATGAAGACTGTTGTTTCCCAACTGTGTAA

RpSPL9 mRNA

ATGAGTTCTGGTTCTGCGACCAAAGCACCACCACCACCACCACCACCACCCTCTTCCTCCTCTGTGCCCAACTCCTCCACTGAGTCCCTTGATGGCTTGAAGTTTGGCCAAAAAATTTATTTTGAGGATGTGAGTGTTGGAGCTAAGGCCAAACCAAGTGGTGGGGCCTCAAAGAAGGGAAGGGGTAGTGCAGTTCATGCAACTCAGCCTCCAAGGTGTCAGGTTGAGGGCTGTAAAGTAGATCTGAGTGGTGCTAAGGCTTACTATTCTAGGCATAAAGTTTGTGGCATGCATTCTAAATCCCCCACTGTCGTTGTCGCTGGTCTGGAACAAAGGTTTTGCCAACAATGTAGCAGATTTCATCAGCTTTCTGAATTTGATCAAGGAAAACGAAGTTGCCGCAGGCGACTAGCTGGCCATAATGAACGTCGGAGAAAGCCCCCACCGTGCTCCTTATTAACCTCACCTTATGCCAGACTTTCTTCACCTATTTTTGATACCAATGGCAGAGGTGGTGGCTTTCTGATGGAATTTGCTTCATACCCAAAGCTTACTCTGAGTAATGAATTGCCAACTCCCAGATCATCTGAGCCAGTTCCTGGTAATCAACCTACAACACTTACCTGGCAGGCGAATATGCAGACACCATCTGACTATTTCCTGCAAGATTCAGTGGGTGTGACAAGCTTCCCTGGTCACAGACATCCTCCAGTGGAAAGTTACACTGAAGTCACAGACTCAAGCTGTGCTCTCTCTCTTCTGTCAAGTCAAACATCATGGGGTTCTAGAAACACCACACCAAGTGTTGAGCTGAACAACTTGTTTAATTTCAATGGGACACTCATGACACAACTTGCTGCATCCTCTCAAGTTGCAGCCATCCATCAACTTCCCAATGCCTCATGGGATTTCAAAGGCATTGATTCTGGTAATTGTTCACCTGAGGTTGTGCCTGATCTCGGTCTGGGTCAGATTTCACAGCCTCTTCATAGCCATCATCCTGGTGAGCTTGGTGTGTCACAGGAAGGCATGAGGCATTACATGGATCTAGGGCAGTCCAGGGCCTGTGAGTCTCCTCACTGGTCACTTTAA

RpSPL10 mRNA

ATGGAGTGGAATGCAAAATCTCTCGGCCAGTGGGACTGGGAGCACTTGTTCTTCTTGAATGCAAAAGCAACAGAAAATTCCAGGTTACAACCTACTGATTGGAGTGGCGAAACAGATCGAGAAATCAATGTTGGAGTGTTGTATCCATCAGGTGGTAGTGGTTGTTCTGGGGTGTCTGAACTAATACATGCTTCTTCCTCAAGGAGCTCAAAATCAGCTTCCAACAATTCATCATCAAATGGGGATAGCAAGACATCTTTGTTAACTTTGGAAGGTTCTCAAGATGATTCAAGTGGTAAGAAAGAATTGTCCAAAGGTGAACCAGTTGAAACTTCTCCAACAGCAGAGCCCTCTTCTGTCTCTGGTGAACCATTGCTCACTCTAAAGCTTGGTAAAAGATTGTACTTTGAGGATGTTTGTGCAGGAAGTGATTCCAAGAAAGCATCTTCTTCTGGGATTCCTATGTCTCGTGGAAAGAAATGTAAATCCATTAGTCAGAACTTGCAACATGCAAGCTGCCAGGTGGAAGGCTGTGGTCTTGATCTCTCATCTGCTAAAGATTACCATCGCAAACATAGAGTTTGTGAAAGTCATTCCAAATCACCTAAGGTGGTTATAGCTGGTTTGGAACGTCGATTTTGTCAGCAATGTAGCAGGTTCCATGCTCTGTCAGAGTTTGATGATCAAAAGAGAAGCTGCAGAAGACGTCTTTTAGATCACAATGCAAGGCGTCGCAAACCTCAGCATGAAGCAGTGCAATTAAATCCATCAGCTTTGTCTTCATCACCCTATGACGGAAGGCAAATAATGAATCCATATGCATTTTCAAGGACTGCTACAAATTTAGCATGGCAAGACATACACAGCAGCAAGCTCCCCCAAACAAAAGATTTTCTTTTGAAGCCTGCAAAAACGTTCAATAAGATGCCAAGTATTGTCACTATGCTTTCTGATGATTCTAGTGGCCTTCTTACATCCAGAGGCATACGGACCAAGAGTATTGTTCCAGGTATTGAAGATCCCACTACCTCGTCTGGTACAAATGCTACACAAGATTTTAGCCGTGCTCTCTCTCTTCTGTCAACCAATTCATGGGGTGCATATGAGACTAGGTCCCTTTCACTAGAACACTCCAACCGGACAACCAGTACCACTCAGTCCATAACACATGCAATGACTCAGCGCTTACCTCTTTCTTCATCAGAATATTGGCACGCTGATCATCAACCGGCCAGCTCCAGCATCTGTATCCCATACTCAGATTGTGATACTAGCACTCGCTTTCAAGACTTTCAGCTGTTGAGCGCACCCTTTGAGTCAGGTTTTCCTTGCAACCAGCTGGATTGA

RpVAL mRNA

ATGGGTTCGGACATTGGTGTTGTCAATGCTTCGTGCGTTCATGAATGGAAGAAGGGGTGGCCTCTGCGATCTGGTGGATTTGCTCAACTTTGCTGCAAGTGCGGATTTATGGAGGAATGGAAGCGGGACATCCTTGATCCTTTATCTGCCTATGAGAGTTCCGTTTTCTGTAATAAATTCCACTGTCAAGAAACTGGTTGGAGGGACTGTAAATTTTGCAACAAGCCTATCCACTGTGGATGCATAGTATCTAGATCTTTGTTTGAGTATCTTGACTTTGGTGGTATAGGTTGTGTTAGCTGTGTAAATACTTCCCTGCTCAGTATGATGAGGAATAATGAAATTCCTAACGGGTCTGGTTCATTGAACCAAACTAATGCAAGGGATAGACATTCTGCTCATTTTGATGGCAGACTGTTTGCGGGTAGTGTTGATGAAGGAAAACTTATGCAATTGTGCAGAGTTGTTGAAGCTAGTGAATTCAGCCGCTGGAATCATAAAGCTCAGAGAGATGGCATGATTAAAAGTAGTGGGCAAAACAGCCAAGAAGTTAAGTGTTCATTCAGGGAAGTGGATACTAGATTTTCAAATGTGATTAAACCATCTGTTCAGTCATTAACATTTGCTACATTAGAAAATAATAGATCACCATGGGAGATTAAAAACATTCATGAGTCAACTGCACAGCCATCTTTGAGTATGTATTTGGGAAACCCTTCAGGGAACCAATCTGTCCCACCTTCTGCTGGAGAGAATGTAGAAGGAAGAGTTCAGGCCAAAGCATCTCCTCCCTTTCATCAAGGGCAAAGATCTCGCCCTATATTGCCCAAACTATTGAAGACTGAGCTTACCATGAATGTGGAAACTGATAAAGGCTCAGTTTTCCAATCACGTATTGCCCGGCCACCAGCTGAAGGGAGGGGCAAGAATCAGTTACTTCCTCGATACTGGCCCAGGATTACTGATCAAGAGCTGGAGCGATTGTCCGGAGATTTGAAGTCCACTGTGGTGCCATTATTTGAGAAGGTGTTGAGTGCCAGTGACGCAGGTCGAATTGGTCGTCTTGTTCTCCCAAAAGCCTGTGCTGAGGCTTATTTTCCTCCTATTTCACAATCTGAAGGTCTTCCTTTGCGGATGCAAGATGTGAAGGGGAATGAGTGGACATTTCAGTTCAGATTTTGGCCTAATAACAACAGTAGGATGTATGTATTGGAGGGTGTGACCCCTTGCATACAGGCCATGCAATTACGTGCTGGTGATACCGTAACATTTAGTCGGATAGATCCTGGGGGAAAACTTGTTATGGGTTTCAGAAAGGCATCAAATTCTATAGATGCACAGGATGCCTCTACATCTGCACAGTCTAATGACATTTCAGCAAAGGGAACCACCTTTTCTGGTGGAACTGAGAATCTGCCATCAGGAAGTAATTATGCTGAACTTCTTCATTCGATGAAAGGGAATGGGGAACCTCACTTGAATGGACTTCCAGAACATCTGCATTTGGGTACTGAAGCTGCTGGTTTGATTAAAACTGAAAATGGTGAGATGACAGACAATCATTCACTGCAGTTACGAATTTCAGTTTTAGAGAAGAAGAGGACTCGCAATATTGGGCCTAAAAGTAAGAGGTTGCTTATTGATAATGAAGATTCTATGGAGTTGAGACTTACATGGGAAGAGGCACAGGACTTGCTTCGTCCACCACCTAGTGTGAAGCCAAGCATCGTCACAATTGAGGACCAAGTATTTGAAGAATACGATGAACCCCCAGTTTTTGGAAAGAGAACAATATTCAGTGCCTGTTCATCTGGGGGGAAGGAACAATGGGCTCAATGTGATGACTGCTCTAAATGGCGAAAGCTGCCAGTTGATGCTCTTCTTCCTCCCAAGTGGACATGTTCTGAAAATGTTTGGGATACAAGCAGGTCTTCATGTTCTGCACCAGAGGAACGAAGTTCAAGGGAATTAGAAAATCTTTTGAAAACCAGCAAAGATTTTAAGAAGCGACGAATAATAGAAAATGGCAAGTCGATCCAAGAACCTGAGCCTTCTGGCTTAGATGCTCTTGCCAGTGCAGCAGTTTTAGGAGAAAATCTTGTTGACCCTACTGAGTCATCAGCTGGAGCCACCACCAAACATCCTAGACACCGTCCTGGCTGTTCTTGCATTGTATGCATTCAGCCCCCAAGTGGAAAGGGAAGACATAAGCCAACATGCACATGCAACGTGTGCATGACTGTGAAGCGCCGGTTCAAAACCCTCATGCTACGGAAGAAGAAACGCCAATCGGAACGTGAAGCAGATGCTGCTGCACAAAAAGATGATCATATTCACCAAAAAGATGAGTCAGATACCAATGGAGGAGCATCAAGAGATGATGATACAAGTCATTTAGGGAAAGAGGGAGGACTAAACAAAGGTCAATCTGAGGTTGGTGAGTCCAGTGCTGGACAAATAGATCTGAATTCTCATCCCAATCGTGAAGACTTGCAAAGGGATATCACAGGACTTAACATGTCAAGTCCTCATCTTGAAACAACAAACCATGAGGTAAGGGAATATATGAATCAAAATGGCTTAAGAAGCTTTAATAATAGTGAAGTGCAGGCTGATCAACATTCTTCTTTGCTCACTCAATCTAATGGAGAAGGTCAGAGATACTTTTCTGATGAAAGATGTTTATCATCATTTATCTGGAACCAGGAAAAAAGAGGTGAGGTACACAGTCATCCCAATCAAAGTGAAAAAAATCAATCATAA
