# Supplementary material for: Effects of the vegetative propagation method on juvenility in Robinia pseudoacacia L
Source: For Res (Fayettev). 2022 Dec 5;2:17. doi: 10.48130/FR-2022-0017 (PMC11524284; doi:10.48130/FR-2022-0017)
Supplement: Supplementary file 1 — Supplementary data to this article can be found online. [file FR-2022-0017-S1.zip › 10.48130_FR-2022-0017-Suppl-FigureS1.docx]

**
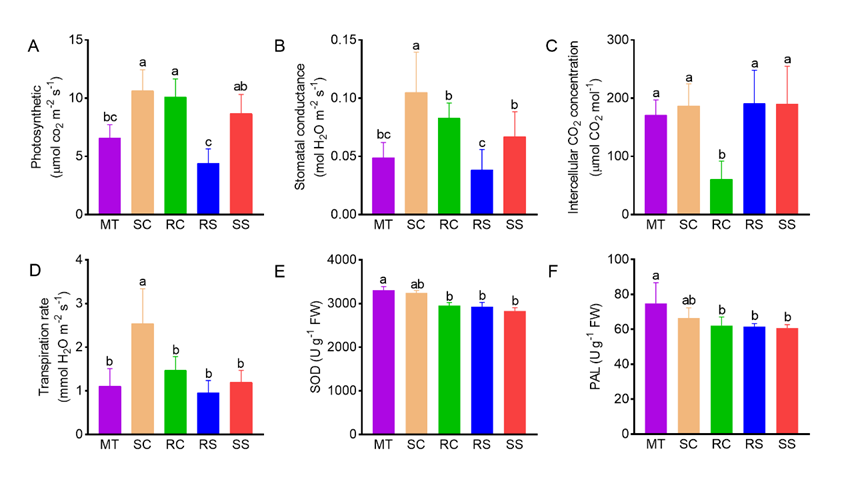
**

**Fig. S1.** Physiological and photosynthetic traits of juvenile plantlets/seedlings and mother trees. (A) Photosynthetic. (B) stomatal conductance. (C) intercellular CO_2_ concentration. (D) transpiration rate. (E) SOD. (F) PAL. Different lowercase letters above the bars indicate significant differences among the plant materials. Abbreviations: SS, seed-derived seedlings; RS, root-sprout plantlets; RC, root-cutting plantlets; SC, shoot-cutting plantlets; MT, mother trees; SOD, superoxide dismutase; PAL, L-phenylalanine ammonia-lyase.
